# Image quality evaluation of real low-dose breast PET

**DOI:** 10.1007/s11604-022-01293-y

**Published:** 2022-05-25

**Authors:** Yoko Satoh, Masamichi Imai, Chihiro Ikegawa, Hiroshi Onishi

**Affiliations:** 1 Yamanashi PET Imaging Clinic, Shimokato 3046-2, Chuo City, Yamanashi Prefecture 409-3821 Japan; 2 Department of Radiology, University of Yamanashi, Shimokato 1110, Chuo City, Yamanashi Prefecture 409-3898 Japan

**Keywords:** Dedicated breast positron emission tomography, ^18^F-Fluorodeoxyglucose (FDG), Real low-dose FDG

## Abstract

**Purpose:**

To evaluate the clinical feasibility of high-resolution dedicated breast positron emission tomography (dbPET) with real low-dose ^18^F-2-fluorodeoxy-d-glucose (^18^F-FDG) by comparing images acquired with full-dose FDG.

**Materials and methods:**

Nine women with no history of breast cancer and previously scanned by dbPET injected with a clinical ^18^F-FDG dose (3 MBq/kg) were enrolled. They were injected with 50% of the clinical ^18^F-FDG dose and scanned with dbPET for 10 min for each breast 60 and 90 min after injection. To investigate the effect of the scan start time and acquisition time on image quality, list-mode data were divided into 1, 3, 5, and 7 min (and 10 min with 50% FDG injected) from the start of acquisition and reconstructed. The reconstructed images were visually and quantitatively compared for contrast between mammary gland and fat (contrast) and for coefficient of variation (CV) in the mammary gland.

**Results:**

In visual evaluation, the contrast between the mammary gland and fat acquired at a 50% dose for 7 min was comparable and even better in smoothness than that in the images acquired at a 100% dose. No visual difference between the images with a 50% dose was found with scan start times 60 and 90 min after injection. Quantitative evaluation showed a slightly lower contrast in the image at 60 min after 50% dosing, with no difference between acquisition times. There was no difference in CV between conditions; however, smoothness decreased with shorter acquisition time in all conditions.

**Conclusions:**

The quality of dbPET images with a 50% FDG dose was high enough for clinical application. Although the optimal scan start time for improved lesion-to-background mammary gland contrast remained unknown in this study, it will be clarified in future studies of breast cancer patients.

## Introduction

^18^F-2-fluorodeoxy-D-glucose (^18^F-FDG) positron emission tomography/computed tomography (PET/CT) is one of the most useful diagnostic imaging tools for cancer. Many studies have demonstrated the efficacy of whole-body FDG-PET/CT in staging or re-staging, monitoring response to therapy, and predicting the prognosis of patients with breast cancer [1–3]. It is important to detect breast cancer at an early stage, when lesions are small, and to accurately determine the therapeutic effect of neoadjuvant chemotherapy on improving prognosis [4, 5].

Because of the limited spatial resolution of whole-body PET/CT to detect small breast cancers, high-resolution dedicated breast PET (dbPET) was developed for the detection of early-stage breast cancers. Previous reports have shown that ring-shaped dbPET can visualize smaller breast cancers better than whole-body PET/CT [6, 7]. In addition, since dbPET has a highly sensitive detector and does not use X-ray CT to perform attenuation correction, it is expected to greatly reduce the radiation dose to patients when it is used alone compared to whole-body PET/CT for local assessment and screening of breast cancer.

Dose reduction related to the use of dbPET based on phantom tests and clinical cases was previously evaluated; the results revealed that by employing 25% of the standard ^18^F-FDG dose, it is possible to obtain a clinically acceptable image quality, and 12.5% of the standard dose results in an image quality that is still sufficient for the detection of lesions [8]. In the aforementioned study, low-dose images were simulated for clinical cases. However, with regard to ethics, previous studies have not evaluated dbPET imaging with real low-dose PET. The simulated low-dose images in the previous studies were obtained with divided full data (i.e., 100% data), full time acquisition (7 min), and at a full dose (3 MBq/kg). While the injected dose for the list mode data cannot be changed, it is possible to shorten the acquisition time and obtain a simulated low-dose image. For example, a “simulated 50% dose image” made from half the full data is in fact obtained in half the acquisition time (3.5 min) at the full dose. Since the phantom is non-living and has no metabolism, the two images can be considered similar, but in humans, where glucose is constantly being metabolized, the two images are not the same. Therefore, the images obtained in this study with full time acquisition and with real low-dose FDG have the true metabolic information, unlike the simulated low-dose images.

To the best of our knowledge, no study has assessed real low-dose FDG dbPET images to reduce radiation exposure. Therefore, dbPET scans were performed with real low-dose FDG, and the clinical feasibility of low-dose dbPET was assessed by comparing low-dose dbPET images with full-dose images.

## Materials and methods

This study was approved by the Ethics Committee of our institution. All procedures were performed in accordance with the ethical standards outlined in the 1964 Declaration of Helsinki and all its subsequent revisions. Informed consent was obtained from all subjects.

### Ring-shaped dbPET scanner

The ring-shaped dbPET scanner (Elmammo, Shimadzu Corp., Kyoto, Japan) comprises 36 detector modules arranged in three contiguous rings and has a diameter of 195 mm, an axial length of 156.5 mm, and a depth-of-interaction measurement capability [9]. The transaxial effective field of- view (FOV) was 185 mm. Each detector block comprises a four-layered 32 × 32 array of lutetium oxyorthosilicate crystals coupled to a 64-channel positron-sensitive photomultiplier tube via a light guide. Attenuation correction was calculated using a uniform attenuation map with object boundaries obtained from emission data [10]. Scatter correction was performed using the convolution–subtraction method [11], with kernels obtained by background tail fitting. The characteristics and standard performance of this scanner have previously been reported in detail [12].

### Imaging

Nine women who met the inclusion criteria (provided signed informed consent, had no history of breast cancer, and were previously scanned by dbPET injected with a clinical ^18^F-FDG dose [3 MBq/kg] within the previous 3 years) were included in this study. The median age of the participants was 49 years, with a range of 41–60 years.

The average and standard deviation of the nine participants’ body weight, injected dose (and difference from theoretical value), and time from FDG injection to the start of the scan (first and second scan) were 52.24 kg ± 6.2 kg, 83.2 ± 10.75 MBq (5.01 ± 4.84 MBq), 59 ± 3 min, and 90 ± 3 min, respectively. Participants were injected with half the clinical dose of FDG and scanned with dbPET. The average interval between the previous full-dose dbPET and this half-dose dbPET was 10.67 months, with a range of 2–29 months.

Three of the women were premenopausal, six were postmenopausal, and none was menopausal when the two dbPET examinations were performed. None of the participants was diabetic. The right and left breasts were scanned by dbPET scanner for 10 min each 60 and 90 min after ^18^F-FDG injection.

### Image reconstruction

All dbPET images were reconstructed using 3D LM-DRAMA with one iteration and 128 subsets. This was based on a previous report in which DRAMA provided excellent signal-to-noise ratio images by one-pass (single iterative) reconstruction and outperformed other iterative reconstruction algorithms, namely OSEM and RAMLA [13, 14]. Attenuation correction was calculated using a uniform attenuation map with object boundaries obtained from emission data [10], and scatter correction was performed using the convolution-subtraction method [11] with kernels obtained by background tail fitting. The voxel size was 0.78 × 0.78 × 0.78 mm^3^.

**Fig. 1:**
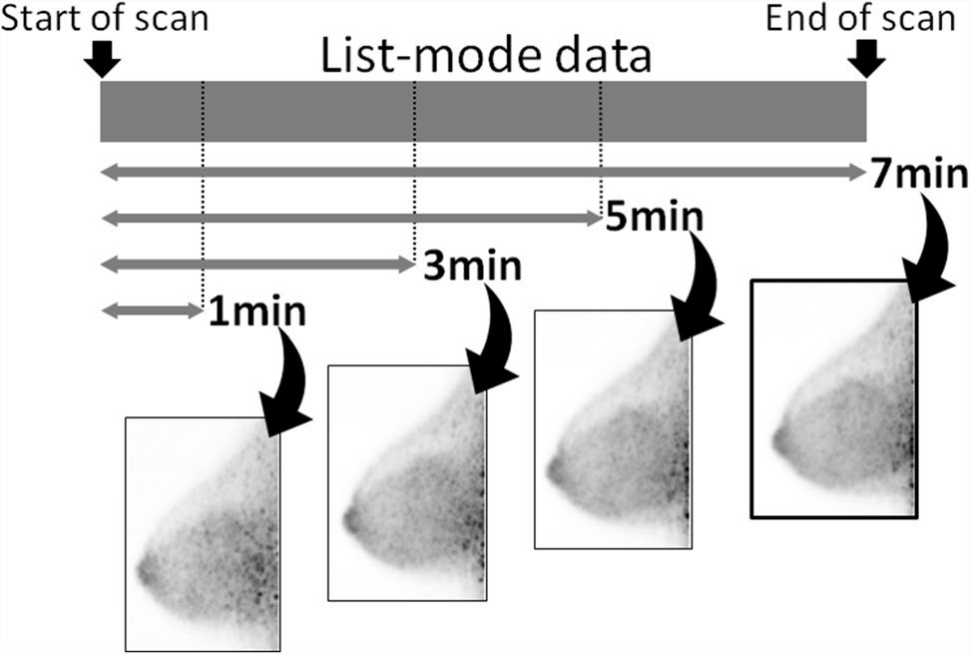
Image reconstruction with divided list-mode data. Reconstructed images from the data acquired for 7 min with full-dose FDG are shown. For injection with half-dose FDG, images were reconstructed from the data acquired for 10 min. FDG, ^18^F-Fluorodeoxyglucose

In this study, only the dbPET images of the right breast were evaluated to avoid the effects of FDG accumulation in the myocardium located outside the FOV on the side of the left breast. To investigate the effect of the scan start and acquisition times on image quality, list-mode data of the right breast were divided into 1, 3, 5, and 7 min (and 10 min with 50% FDG injected) from the start of acquisition and were reconstructed (Fig. 1).

### Analysis of image quality

First, all reconstructed dbPET images using the medio-lateral and cranio-caudal maximum intensity projection images were visually evaluated by an experienced nuclear medicine physician and two experienced PET technologists blinded to the reconstruction settings. The images were displayed on an inverse grayscale with a standardized uptake range of 0–4. All reconstructed mages were assessed for contrast between the mammary gland and fat, as well as smoothness (low noise) using a four-point scale (ranging from a score of 0 [not acceptable for diagnosis] to 3 [good]). The final score was the mean of the scores from three readers according to previous reports [14, 15].

**Fig. 2:**
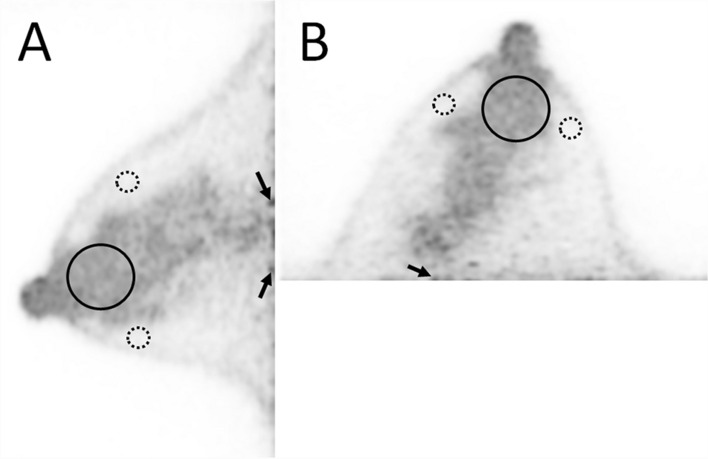
Placement of VOI (volume of interest) in the measurement of dbPET images. For the calculation of contrast and CV, one spherical VOI that was as large as possible was placed in the FDG uptake of the mammary gland (solid black line) and four spherical VOIs of 1 cm diameter in the fat (dotted black line). Sagittal (**a**) and transaxial (**b**) dbPET images

Second, for quantitative evaluation of the clinical images, a volume of interest (VOI) that was as large as possible was placed in the mammary gland, avoiding the nipple, skin, and noise at the edge of the FOV on the chest wall side (Fig. 2). In addition, four spherical VOIs of 1 cm diameter were placed per breast in the fat, avoiding the mammary gland and skin (Fig. 2). The contrast between the mammary gland and fat (contrast) and the coefficient of variation of the mammary gland (CV) between each image were statistically compared. Contrast is the ratio of the mean of standardized uptake values (SUV_mean_) of all VOIs placed on the mammary gland and fat. CV is the ratio of the standard deviation to the mean and indicates the degree of variation of uptake intensity relative to the mean. A higher CV means a noisier image with much noise.

The contrast and CV were calculated according to the following equations:

$${\text{Contrast = }}\frac{{{\text{average}}\,{\text{SUVmean}}\,{\text{of}}\,{\text{the}}\,{\text{mammary}}\,{\text{gland}}}}{{{\text{average}}\,{\text{SUVmean}}\,{\text{of}}\,{\text{fat}}}}$$

$${\text{CV = }}\frac{{\,{\text{SD}}\,}}{{{\text{SUVmean}}\,{\text{of}}\,{\text{the}}\,{\text{mammary}}\,{\text{gland}}}}$$

where SD is the standard deviation of SUVs for all voxels within the VOI of the mammary gland.

All quantitative processes were performed using in-house-modified Metavol [16]. The ICCs of contrast and CV calculated from the VOIs independently placed by the two readers (YS and MI) were 0.88 and 0.59, respectively, indicating that the reproducibility of the measurements was GOOD for contrast and FAIR for CV. The averages of these indicators for the two readers were used in the statistical analysis of this study.

### Statistical analysis

Visual scores for different scan start times and acquisition times were compared with those of standard scans using the Wilcoxon paired ranked-sum test. In addition, contrast and CV of the images acquired from 90 min after injection of the full dose and 60 or 90 min after injection of the half dose were compared in all three pairs of groups. A *p* < 0.05 was considered statistically significant. JMP^®^ version 16 (SAS Institute Inc., Cary, NC, USA) was used for all statistical analyses.

## Results

**Fig. 3:**
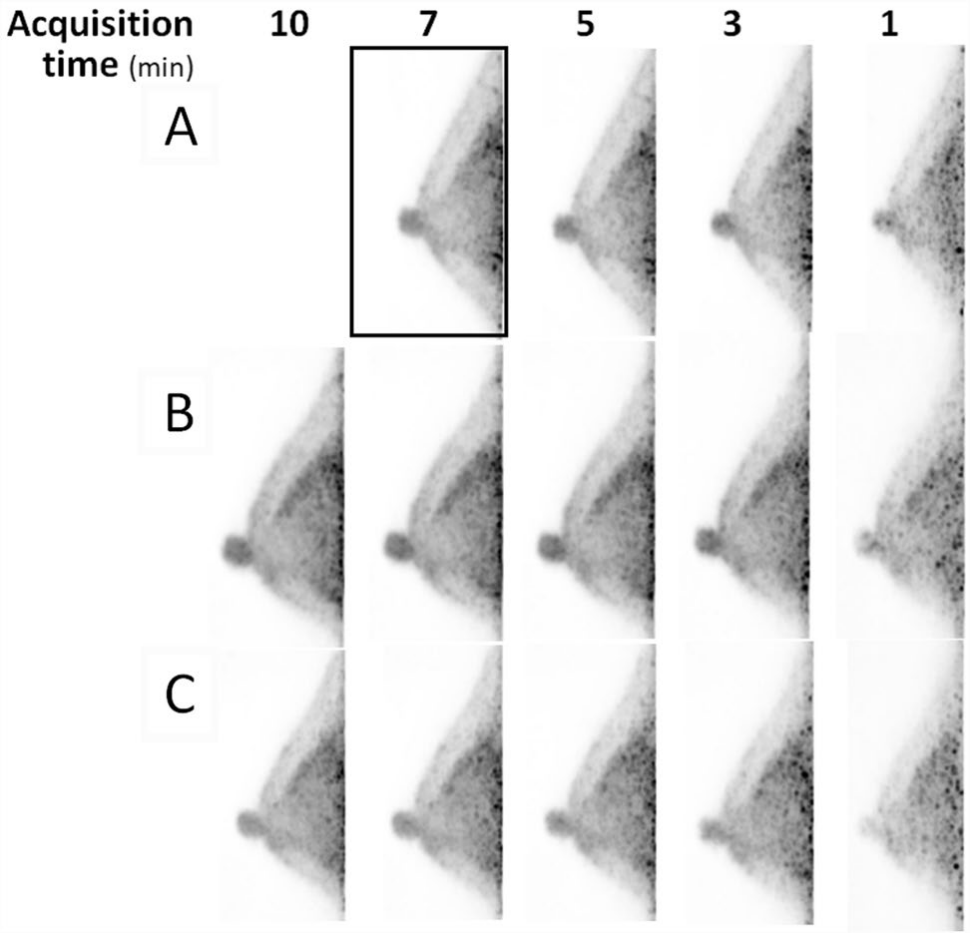
Mediolateral dbPET images of a 50-year-old woman after injection with full-dose (**a**, 3 MBq/kg) and half-dose FDG injection (**b** and **c**, 1.5 MBq/kg). Reconstructed dbPET mages from data acquired for 7 min from 90 min after full-dose FDG injection (**a**) and 10 min from 60 min (**b**) and 90 min (**c**) after half-dose FDG injection. FDG, ^18^F-Fluorodeoxyglucose

Figure 3 shows an example of the dbPET imaging results.

**Fig. 4:**
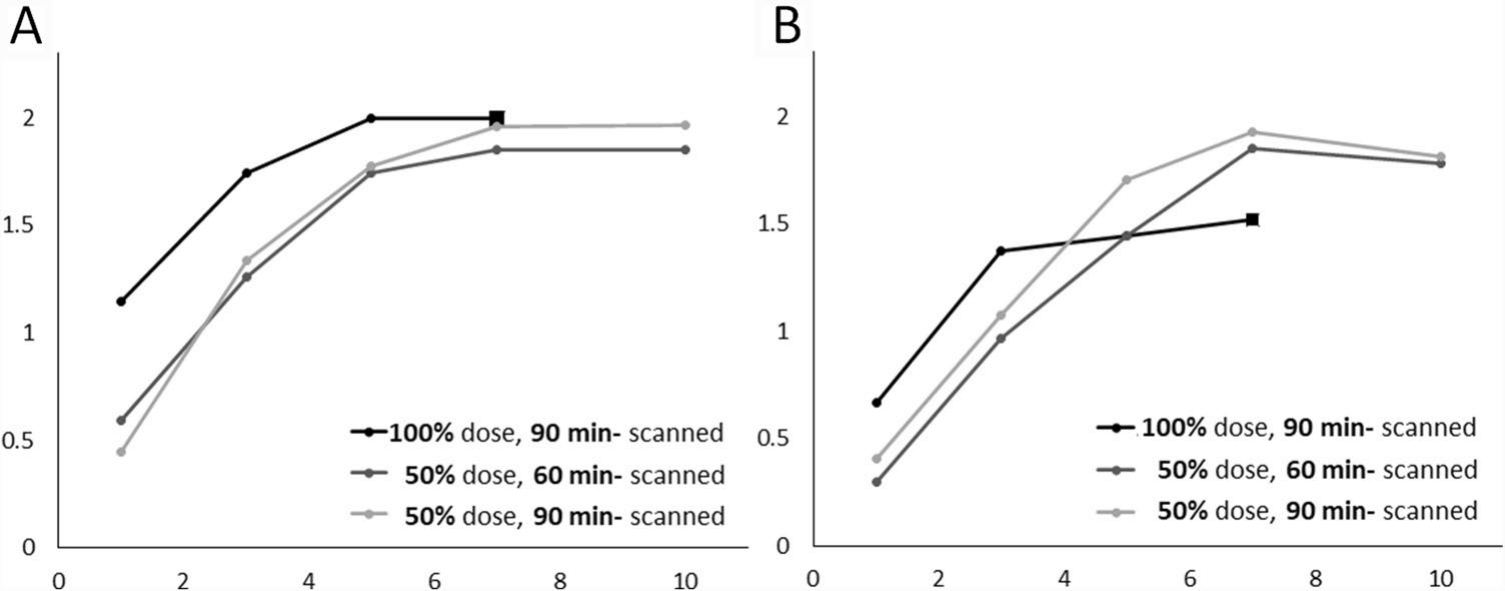
Image quality ratings of the contrast between the mammary gland and fat (**a**), as well as image smoothness (low noise) (**b**). The x-axis represents the data acquisition time (min) used for image reconstruction and the y-axis represents the mean score of the raters. The doses and the scan start time were 100% dose and 90 min (black line), 50% dose and 60 min (dark gray), and 50% dose and 90 min (light gray)

Table 1 shows the results of the visual evaluation of dbPET images with different doses of injected FDG, scan start times, and acquisition times. Rating the contrast between the mammary gland and fat, as well as the smoothness, showed significant differences among several reconstructions (Fig. 4; Table 1). The contrast of dbPET images acquired for 7 min with a 50% dose was the same as that of the images acquired with a 100% dose, while the smoothness was higher. The contrast and smoothness of dbPET images acquired with a 50% dose were not significantly different between 7-min and 10-min acquisition times. Even with a 100% dose, both the contrast and smoothness of dbPET images decreased when the acquisition time was less than 3 min.

**Table 1:** Image quality ratings and *p* values for comparison with the clinical standard scan†

Injected dose	Scan start time^a^ (min)	Acquisition time.^b^ (min)	Contrast			Smoothness (low noise)		
			Mean	SD	*p*	Mean	SD	*p*
Full (3 MBq/g)	90	7	2	0		1.52	0.51	
		5	2	0	1	1.44	0.51	0.3466
		3	1.74	0.45	0.0431*	1.37	0.49	0.0353*
		1	1.15	0.46	< 0.0001*	0.67	0.55	< 0.0001*
Half (1.5 MBq/g)	60	10	1.85	0.36	0.1038	1.78	0.51	0.0881
		7	1.85	0.36	0.1038	1.85	0.36	0.0085*
		5	1.74	0.45	0.0653	1.44	0.58	0.6224
		3	1.26	0.59	0.0009*	0.96	0.59	0.0103*
		1	0.59	0.50	< 0.0001*	0.30	0.47	< 0.0001*
	90	10	1.96	0.19	0.3466	1.81	0.48	0.0864
		7	1.96	0.19	0.3466	1.93	0.27	0.0054*
		5	1.78	0.42	0.0497*	1.70	0.47	0.2145
		3	1.33	0.48	0.0001*	1.07	0.55	0.0005*
		1	0.44	0.51	< 0.0001*	0.41	0.64	< 0.0001*

*FDG* 18F-Fluorodeoxyglucose

^†^Clinical standard scan: a scan image acquired for 7 min from 90 min after full-dose injection was the reference image for comparisons with other images

^a^The time in minutes from intravenous FDG injection to the start of the dbPET scan

^b^The number of minutes of the list-mode dbPET data from the start of the dbPET scanning to be used for image reconstruction

**Fig. 5:**
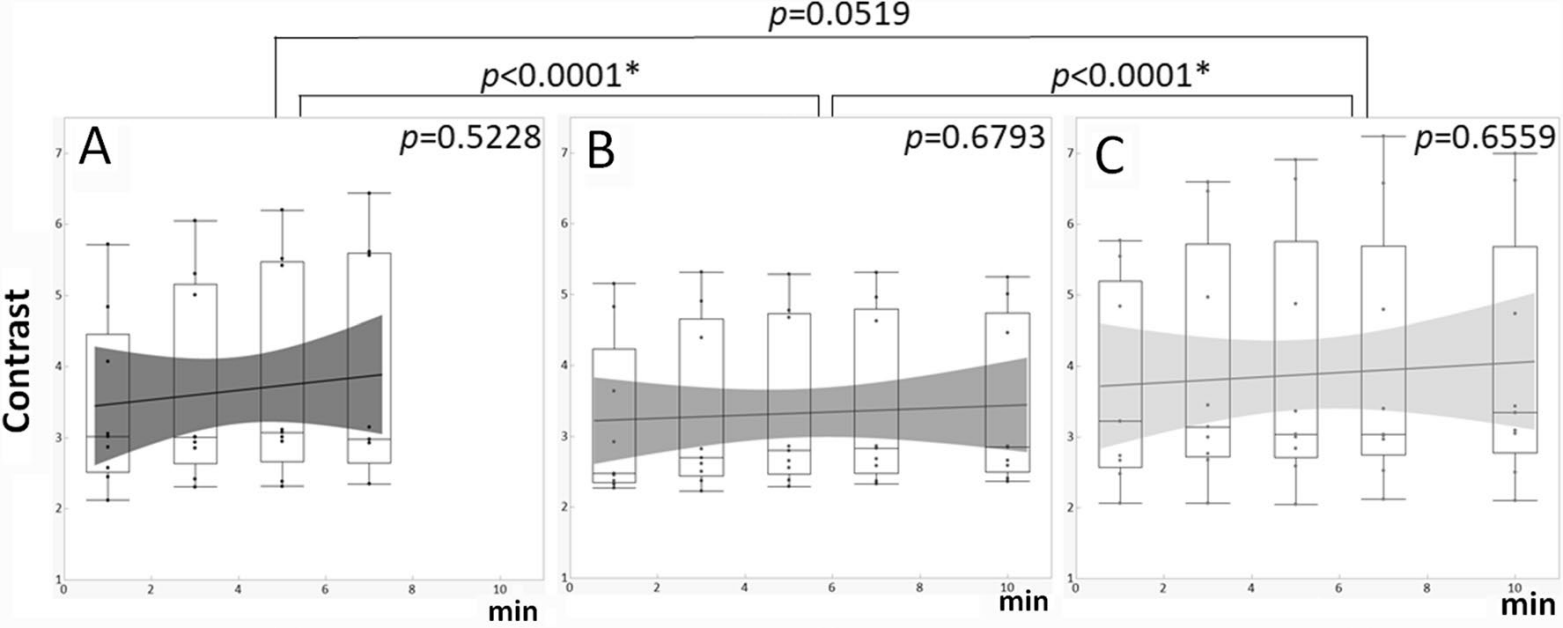
Comparison of contrast between the mammary gland and fat among dbPET images with different injection doses and scan start times. Full dose (**a**) and half dose with a scan start time from 60 min (**b**) and 90 min (**c**). The x-axis represents the data acquisition time (min) used for image reconstruction. The black lines in the graphs indicate regression lines and the gray bands indicate confidence intervals

**Fig. 6:**
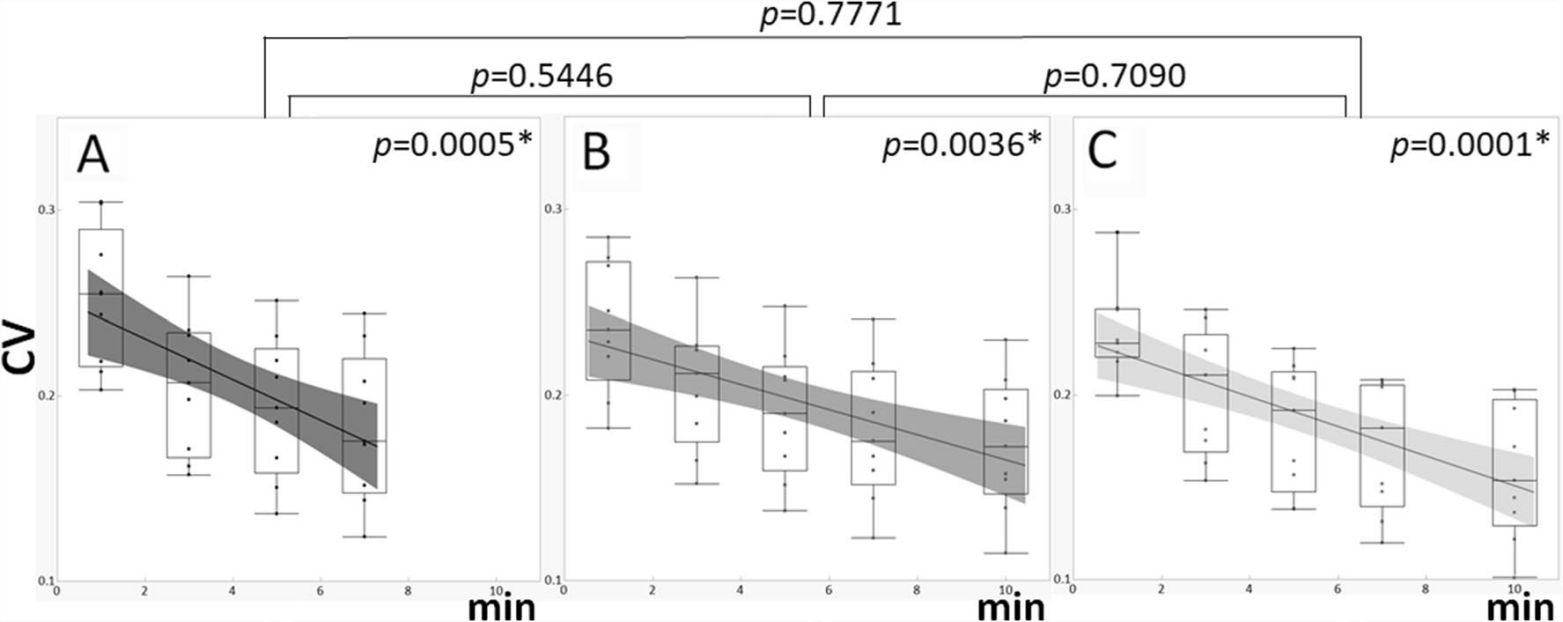
Comparison of coefficient of variation of mammary gland among dbPET images with different injection doses and scan start times. Full dose (**a**) and half dose with a scan start time from 60 min (**b**) and 90 min (**c**). The x-axis represents the data acquisition time (min) used for image reconstruction. The black lines in the graphs indicate regression lines and the gray bands indicate confidence intervals

The contrasts of the images acquired from 90 min after injection of a 50% dose and a 100% dose were comparable, while that of the images acquired from 60 min after injection of a 50% dose was slightly lower (Fig. 5). There was no difference in contrast by acquisition time. CV, on the other hand, increased with shortening of the acquisition time in all conditions, indicating decreased smoothness (increased noise), but there was no difference between conditions (Fig. 6).

## Discussion

This study assessed whether 50% of the dose for whole-body FDG PET/CT provided sufficient dbPET image quality. When the dose is reduced, the absolute amount of FDG that accumulates in the breast reduces compared to when a full dose is used. This implies that the number of annihilation gamma rays from the positrons that accumulate in the breast, which can be measured by the dbPET detector, reduces. Therefore, an emission scan was acquired for 10 min, which was longer than the usual 7 min in this study. As a result, even with the 7 min acquisition that is generally used, the contrast between the mammary gland and fat was almost the same and the smoothness improved. These did not change in the dbPET images with extended acquisition up to 10 min. Therefore, extending the acquisition time was considered unnecessary, even when half the dose of FDG was injected.

When a 50% dose of FDG was injected, there was no difference in contrast and smoothness between the images scanned from 60 and 90 min after injection. Previous studies using whole-body PET/CT reported that SUVs of breast cancer lesions on images of early scans 60 min after FDG injection were higher than those on images of delayed scans 120 min after injection [17, 18]. This is because the contrast between the lesion and the background increases due to the increase in FDG accumulation in breast cancer lesions over time as well as the washout of the physiological accumulation in normal tissues, such as the mammary gland. The physiological uptake of FDG in the normal mammary gland varies according to the menstrual cycle [19], and the uptake to the normal mammary gland in the early scans is washed out in the delayed scans, improving the detectability of breast cancer with low FDG uptake [17]. Since positron-emitting nuclides emit high-energy gamma rays, subjects injected with a PET tracer should wait in a radiation-controlled area until scanning begins. Whole-body PET/CT scans are usually performed from 60 min after FDG injection for about 20–25 min. Subsequently, a dbPET scan is performed approximately 90 min after FDG injection [6, 20]. However, the current 90-min waiting time for a dbPET scan would be too long. Especially in breast cancer screening for healthy women, it is necessary to shorten the time spent in the hospital to reduce the physical burden and the risk of infection for the person going to be examined. Our results showed that it is possible to reduce the waiting time from 90 to 60 min with dbPET examination alone. However, the study only included normal cases without lesions, such as intraductal papillomas or fibroadenomas, which can be positive on dbPET images. Conversely, one participant (in her 40 s) was known to have a small cyst and another (in her 60 s) had a fibroadenoma, both noted on ultrasound, but they were not visualized on dbPET images. If positive lesions could be observed on dbPET, the impact of dose reduction on visualization of small breast lesions using measurements such as lesion-to-background uptake ratio could be confirmed. This is one of the issues to be considered in the future.

It was reported that PET image quality could be maintained even with reduced injection dose using simulated low-dose PET images reconstructed from divided list-mode data [8]. Furthermore, it has been reported that artificial intelligence technology could theoretically make the image quality of low-dose PET images equivalent to that of normal-dose images [21]; however, it has not yet been put to practical use in clinical practice. In contrast, since the real dose was reduced to half the conventional dose in this study, it is expected that our results can contribute to the practical application of low-dose PET.

dbPET images of the left breast were excluded from this analysis because myocardial FDG accumulation does indeed affect the dbPET image of the left breast. Moreover, the degree of myocardial accumulation varies from day to day, even in the same patient. Since the aim of this study was to reduce exposure as much as possible, no PET/CT imaging was performed. Therefore, it could not be ascertained whether the accumulation of FDG in the myocardium of the participant in this real low-dose FDG study was lower or higher than it was in the previous PET examination. This means that if there was a difference between the previous full-dose and current low-dose dbPET images of the left breast, we would not be able to distinguish whether it was due to myocardial accumulation or a reduced dose. Nevertheless, the effects of radioactivity outside the field of view, such as in the myocardium, is an important issue that should be urgently explored.

Annual screening with contrast-enhanced MRI for high-risk breast cancer is now recommended [22, 23]. Although the breast cancer detection ability of breast PET is similar to that of MRI, dbPET has a disadvantage of radiation exposure. However, the advantages of dbPET examination are as follows: (1) there are no side effects due to the absence of a contrast medium, (2) the imaging time is less than 15 min for both breasts, which is shorter than the total MRI examination time, and (3) claustrophobic patients can tolerate the examination as they are not required to lie prone in an enclosed space. Therefore, further reducing exposure to dbPET examination may make it more convenient than MRI as a tool for frequent breast cancer screening. Another disadvantage of dbPET is that the mammary gland near the chest wall may sometimes be outside the FOV of dbPET compared to MRI. A new ring-shaped PET scanner with a larger detector diameter than ours has been developed [24]; it may solve this issue, i.e., reduce blind areas.

Our results only showed that a half-dose dbPET scan, which halves the exposure compared to a full-dose dbPET scan, provides good image quality for diagnosis. The benefit of mammography screening, which is widely performed with public funds, is a reduction in mortality from breast cancer among women screened and the associated economic benefits, whereas the disadvantage is the induction of secondary cancers due to radiation exposure and the burden of public examination costs. Therefore, it is easy to draw conclusions because there are relatively few factors to be considered in mammography screening.

In contrast, the disadvantages of breast cancer screening using dbPET include a high cost and the induction of cancers in other organs in addition to breast cancer, since the exposure is over the whole body. Therefore, it would be impossible to conclude on the ideal extent of exposure reduction.

A previous report from Japan on cancer screening using PET indicated that the risk–benefit break-even point for women in terms of radiation exposure was in the 50 s [25]. Therefore, in older women, it may not be necessary to reduce the dose at the risk of reducing the detection rate of breast cancer. Since a capacity–response relationship between radiation dose and morbidity risk has also been reported in younger, BRCA1/BRCA2 mutation carriers, a highly accurate examination with very few false positives or false negatives should be used. In this regard, the contribution of the low-dose dbPET scan can be expected.

Recent studies on dose reduction in PET have reported on ultralow dose using artificial intelligence [21, 26]. Ultralow doses, such as 1/100 of the conventional dose, would certainly have a significant impact. However, it would be difficult to accurately administer such extremely low doses of FDG using the currently popular automatic dosing devices. Although no conclusion can be made yet, we need to continue our efforts to reduce unnecessary exposure while maintaining image quality.

Our study had several limitations. First, the number of participants was small and their age distribution was narrow. Particularly, few participants were premenopausal women, who are more likely to be candidates for stand-alone dbPET (e.g., high-risk screening/uncertain screening mammograms due to dense breasts). The small proportion of premenopausal women was mainly due to the inclusion of women who had undergone full-dose PET/CT and dbPET within 3 years and without a history of breast cancer. The number of premenopausal women who could have opted for screening with self-funded PET was relatively small. Furthermore, the retrospective nature of the study precluded the inclusion of a sufficient number of premenopausal women. Although the sample size was small and may be considered only informative, it was confirmed that real low-dose FDG provided sufficient image quality for clinical use. This may mean that basic knowledge has been obtained for planning larger clinical trials with a larger number of subjects. Second, our study population did not include patients with small breast cancers, which should be evaluated to determine whether low-dose PET images can sufficiently detect the lesions. However, for breast cancer patients, whole-body PET/CT scans should not be omitted for the diagnosis of metastasis to the lymph nodes and distant organs. Therefore, it may be difficult to realize a study comparing two dbPET scans from 60 and 90 min after FDG injection. Third, this study used a 50% FDG dose, a higher dose than what was used for the previously reported simulated low dose images. This is because it was difficult to accurately prepare and inject a very small amount of FDG under the current clinical conditions. The feasibility of a dbPET scan with a smaller dose of FDG should also be evaluated to further reduce the exposure dose. Fourth, the left breast was not analyzed in this study. The effect of out-of-field radioactivity, such as due to FDG accumulation in the myocardium, on the image quality of such proximal PET systems is an issue that should be evaluated.

In conclusion, the quality of dbPET images with a 50% FDG dose was high enough to be suitable for clinical application. Although the waiting time after FDG injection can be reduced from 90 to 60 min, it is better to start the scan 90 min after injection, as the contrast between the lesion and the normal mammary gland will be clearer.
